# Reflex Nervous Disturbances

**Published:** 1881-05

**Authors:** Robt. W. Westmoreland

**Affiliations:** Atlanta, Ga.


ATLANTA
Medical and Surgical Journal.

VOL. XIX.] MAY—1881. [No. 2

### Original Communications.

### REFLEX NERVOUS DISTURBANCES.

BY ROBT. W. WESTMORELAND, M.D. ATLANTA, GA.

In no department of medicine is there a more fruitful field for theory than that pertaining to diseases of the nervous system. The prevailing penchant is directed to neuro-pathology, and he is an exception to the general rule who has not one of the "standard" shocking machines in his office with which to torture each patient who may have managed to resist the assault of acological therapeusis. A new book, by some splendid foreign genius, on neuro-pathology, is published by some enterprising translator, and the thousands who read it directly devote their attention to the task of working every symptom, in each individual case, that may come under their observation, into a manifestation of nervous disease.

This mania for refering almost every disturbance of the human body directly to the nervous system presents to the student books, for instance, on *neurasthenia*, where every disorder, even the most minute, is referred, primarily, to disturbance in the nerves as its origin. Beautifully classified under this name of *neurasthenia* we have *astrophobia*, or fear of lighting, *topophobia*, fear of places (with specific sub-divisions) and many others of like character, which are intended to perfect the student in the principles of neuropathy.

Amidst all this enthusiastic nonsense occasionally substantial facts may be arranged into practical suggestions. Those diseases dependent upon an excitation of the reflex function of the spinal cord possess an importance to the general practitioner that should not be neglected. With a full knowledge of the details of reflex action and reflex irritability much will be made clear that would otherwise be in a manner incomprehensible.

The spinal cord does not possess any power of automatic action like the higher nerve centres, but impressions are received and transmitted through the gray matter, subject, more or less, to the influence of the superior centre—the brain. Under circumstances of health, this reflex function is inhibited, to a certain extent, by the commanding influence of the will, but under the excitement or irritation of disease the brain loses its influence, and may even be made to suffer by the train of morbid changes set up. In these instances the spinal column is said to be in a state of polar excitement which is kept up, and it may be increased by the peripheral disturbance.

Take, for instance, a female suffering from a uterine disease of long standing. The original inflammation has resulted in displacement, engorgement and an almost complete change of function and arrangement of the organs of gestation. From the nature of things this is certain to prove a source of continuous irritation so long as this condition of things lasts, and this unceasing flood of morbid stimulus to the spinal centre is certain to disturb and derange the equilibrium of its function.

This alteration or excitation of reflex function manifests itself in almost innumerable ways, and may assume a functional or even an organic character. The train of symptoms thus produced is usually designated "hysterics," and, although a term peculiarly offensive and obnoxious to the majority of females, is yet, in such instances as the one just presented, certainly no misnomer.

We recollect to have heard a physician state that when called on to treat a colored person, unless there were very decided reasons for a contrary course, his plan was, without much search, to adopt anti-syphilitic measures. This course, he remarked, would be attended more frequently with success than failure, so far as propriety of diagnosis was concerned. In like manner we would feel almost as secure in the administration of anti-spasmodics and remedies calculated to lessen reflex excitability to women who were the subjects of uterine diseases, and who suffered, at the same time, from some nervous trouble of apparently obscure origin.

Let no practitioner who may conclude on a diagnosis of "hysterics" be so thoughtless as to announce his conclusion to his patient. For, as a certain result, where he is not unceremoniously dismissed, he will surely incur the decided opposition of the sufferer.

The treatment consists in administering such remedies as will reduce the reflex excitement, and, at the same time, control the secondary difficulty. For most cases bromide potassium will be found sufficient to control the reflex, while local treatment is going on for the cure of the uterine disorder. The secondary disturbance will be found to be of a functional character and very seldom require any special treatment, but will disappear gradually as the primary affection is improved. The main object is to cure the uterine disease, and our principal effort should be in this direction. Until we are successful at this point we can not hope for any permanent good for any series of remedies applied to the general nervous disturbance.

Another most important illustration of reflex irritability consists in the train of symptoms produced by preputial adhesions and deformities in male children. As a result of the nervous disturbance set up by this form of peripheral irritation complete paralysis of the lower extremities may occur. Besides this extreme effect, we often see the minor manifestations of the nervous disturbance arising from the same cause, and assuming the appearance of idiopathic local diseases. A young boy came under our observation who suffered from a most distressing cough, and palpitation of the heart; after failing to demonstrate any other cause for the difficulty, the prepuse was examined and found to be adherent almost the entire circumference of the *corona glandis.* The breaking up of this adhesion resulted in complete cure; the boy gaining flesh and a rosy color rapidly.

Again we may have "seat worms" or the *lumbricoides*, as a potent source of reflex disturbances in children, in which the train of constitutional symptoms will entirely and rapidly disappear after removal of the exciting cause in the rectum.

In the treatment of these morbidly reflex phemomena, remedies directed to the constitutional derangement will rarely exert any permanent benefit. As soon as the immediate effect of the remedy is exhausted, the same train of symptoms will almost invariably reappear. To remove the cause is the prime, and in fact the only, reliable indication. Then if the condition is not of too long duration the nervous element of the trouble will disappear defore the gradual effort of nature.

